# Genomic regions associated with spot blotch resistance in elite barley breeding populations

**DOI:** 10.1007/s11032-025-01537-5

**Published:** 2025-01-16

**Authors:** Dipika Roy, Eric Dinglasan, Ryan Fowler, Greg Platz, Reg Lance, Lisle Synman, Jerome Franckowiak, Lee Thomas Hickey, Kai Voss-Fels, Hannah Robinson

**Affiliations:** 1 Queensland Alliance for Agriculture and Food Innovation, The University of Queensland, St Lucia, QLD 4072 Australia; 2 Queensland Department of Agriculture and Fisheries, Hermitage Research Facility, Warwick, QLD 4370 Australia; 3 Department of Agronomy and Plant Genetics, University of Minnesota, St Paul, MN 55108 USA; 4 Institute for Plant Breeding, Hochschule Geisenheim University, Geisenheim, Germany

**Keywords:** Spot blotch, Resistance, Genome-wide association study, Haplotype, Barley

## Abstract

Spot blotch (SB), a prevalent foliar disease of barley, is caused by the hemibiotrophic fungal pathogen *Bipolaris sorokiniana*. Predominately occurring in humid growing regions worldwide, SB can result in yield losses of up to 30%. Genetic resistance remains the most effective strategy for disease management; however, most Australian barley cultivars exhibit susceptibility despite the previous identification of major resistance loci. This study investigates the genetic architecture underlying spot blotch resistance within an Australian barley breeding program. Resistance was assessed at both the seedling and adult growth stages using a single conidial isolate (SB61) across two consecutive years. A total of 337 barley lines were genotyped with 16,824 polymorphic DArT-seq™ markers. Two mapping approaches were employed: a single-marker genome-wide association study (GWAS) and a haplotype-based local genomic estimated breeding values (Local GEBV) approach. Both methodologies identified two major resistance-associated regions on chromosomes 3H and 7H, effective across growth stages. Additionally, the haplotype-based Local GEBV approach revealed resistance-associated regions on 1H, 3H, and 6H that were not detected by GWAS. Haplotype stacking analysis underscored the critical role of the 7H region for adult-plant resistance when combined with other resistance haplotypes, suggesting significant gene-by-gene interactions and highlighting the complex, quantitative nature of spot blotch resistance. This research confirms the presence of key resistance loci within Australian barley breeding populations, provides novel insight into the genetic architecture of spot blotch resistance, and emphasises the potential to enhance resistance through haplotype stacking and whole-genome prediction approaches.

**Supplementary Information:**

The online version contains supplementary material available at 10.1007/s11032-025-01537-5.

## Introduction

The hemibiotrophic fungal pathogen *Bipolaris sorokiniana* (teleomorph: *Cochliobolus sativus*) causes spot blotch, a foliar disease that significantly impacts barley (*Hordeum vulgare* L.), leading to yield losses of up to 30% *(*Kaur et al. 2022). The disease predominantly occurs in warm and humid growing regions worldwide, and its prevalence is expected to expand due to global climate changes (Bastas 2022; Martínez et al. 2022). This poses a significant challenge to the global barley industry, necessitating effective disease management strategies. Among these, deploying cultivars with robust genetic resistance is considered the most sustainable and economically viable approach (Leng et al. 2020).

The pathogen exhibits high genetic diversity and evolving virulence (Meldrum et al. 2004). Earlier studies on virulence patterns demonstrated that most Australian barley cultivars are susceptible, with only four out of fifteen showing resistance (Meldrum et al. 2004). While quantitative trait loci (QTL) mapping has been instrumental in identifying resistance loci in research populations (Lander and Botstein 1986; Bilgic et al. 2005; Roy et al. 2010), limited efforts have focused on breeding populations. To date, three resistance genes have been fine-mapped. The *Rcs5* gene, associated with all-stage resistance, was mapped to chromosome 7H using the Steptoe × Morex doubled haploid population (Steffenson et al. 1996; Bilgic et al. 2006). The *Rcs6* gene, located on chromosome 1H, was identified in the Calicuchima × Bowman doubled haploid population and confers both all-stage and adult-plant resistance (Bilgic et al. 2006). Notably, the susceptibility allele *Scs6*, dominant and contributed by the cultivar Bowman, was later characterised at the same locus (Leng et al. 2020). Additionally, an interval on the short arm of chromosome 6H, designated *Rbs7*, was consistently detected and contains a small number of candidate genes (Wang et al. 2017, 2019).

In recent years, genome-wide association studies (GWAS) have expanded our understanding of spot blotch resistance. For instance, analysis of the USDA barley core collection, comprising 1,480 accessions, identified six novel QTL associated with resistance on chromosomes 1H, 2H, 3H, 6H and 7H (Wang et al. 2017). Notably, the QTL on chromosome 3H was also detected in the ICARDA spring barley collection (Gyawali et al. 2018), underscoring its global significance. Additional GWAS in ICARDA collections revealed four novel QTL conferring all-stage resistance and nine QTL associated with adult-plant resistance in Morocco and India, respectively. The *Rcs5* region was consistently detected across growth stages and locations, highlighting its importance (Gyawali et al. 2018). Similarly, studies on high-input barley lines identified both novel and previously reported QTL (Visioni et al. 2020). These findings collectively underscore the quantitative nature of spot blotch resistance, suggesting that robust resistance likely requires the combination of multiple small- and large-effect QTL.

The complex genetic architecture of spot blotch resistance poses significant challenges for breeding resistant varieties. For example, the *Rcs6*/*Scs6* locus is tightly linked, yet not allelic, to *Mla8*, a major gene conferring resistance to powdery mildew (Leng et al. 2020). Additionally, the *Rcs5* gene is closely linked and in repulsion with *Rph23*, an adult-plant resistance gene for leaf rust (Ziems et al. 2023). Effective breeding strategies must carefully consider the interplay of these resistance and susceptibility alleles to optimise outcomes. This highlights the need for continuous identification of resistance loci, particularly those with minimal pleiotropic effects. Conventional single-marker GWAS and QTL mapping approaches often overlook linkage and practical applications in breeding programs.

In this study, we evaluated two Australian elite breeding populations for spot blotch resistance across growth stages. Advanced haplotype-based mapping approaches were used to identify chromosomal regions associated with susceptibility within the breeding program. The insights gained provide a foundation for improving genetic resistance and inform strategies for practical application in a breeding program.

## Materials and methods

### Barley breeding populations

A panel of 340 elite two-row spring barley lines from the Australian Northern Region Barley breeding program (NRBBP) including a small sub-set of Australian commercial cultivars (Supp. Table 1) were investigated in this study. Two distinct groups of materials were designated as breeding population one (BP1), which consisted of 141 lines sampled from Stage 2 of the NRBBP in 2012, and breeding population two (BP2), which consisted of 232 lines sampled from Stage 2 of the NRBBP in 2013 (Table 1). A total of 33 lines were in common across BP1 and BP2.

**Table 1:** Summary of the barley breeding populations assessed for spot blotch disease resistance

Breeding population	Year	Environment	Growth stage	Genotypes
1	2012	Glasshouse, HRF^a^, Warwick	Seedling	141
1	2012	Field, RRF^b^	Adult	132
2	2013	Glasshouse, HRF^a^, Warwick	Seedling	231
2	2013	Field, RRF^b^	Adult	232

^α^ All seedling assessments were performed at Hermitage Research Facility, Warwick (HRF)

^b^ All field experiments were conducted at Redlands Research Facility (RRF)

### Pathogen materials

The breeding populations were assessed for resistance to *Bipolaris sorokiniana* at both seedling (all stage) and adult plant stages, using pathotype SB61 at the Queensland Department of Agriculture and Fisheries (QDAF) in the Hermitage Research Facility (HRF), Warwick and Redlands Research Facility (RRF), Cleveland, Queensland, Australia respectively. SB61 is well described and prevalent throughout the growing region (Meldrum et al. 2004) and was originally collected on 2/10/1998 from Monto in Queensland.

### Fungal inoculum preparation

To prepare conidia for inoculation, infected leaves (SB61) were cut into 2 cm segments, heat-shocked in a 40 °C water bath for 3 min, and placed in petri dishes with moistened filter paper. The dishes were incubated at 19 °C with 12-h diurnal light using blacklight blue and white fluorescent tubes to induce sporulation. Single conidia were transferred to Starch Nitrate agar (SNA) media under laminar airflow using a sterilised needle. Plates were incubated in the dark at 25 °C (± 1 °C) for 7–9 days to promote mycelia growth.

For sub-culturing V8 agar media (20% v/v V8 juice, 2 g/L CaCO₃, 15 g/L agar, q.s. to 2 L with distilled water) was prepared. Mycelia from SNA plates were transferred to V8 agar plates using a sterilised 4 mm hole punch and incubated in the dark at 25 °C (± 1 °C) for 9–12 days. Conidia were dislodged by adding 5–10 mL distilled water to the plates and wiping with camel-hair brush. The spore suspensions were filtered through a fine mesh and diluted with Tween®- water (three drops Tween®20 per 100 mL) to a concentration of 10,000 conidia/mL, confirmed with a haemocytometer.

### Screening for seedling-plant resistance in the glasshouse

BP1 and BP2 were evaluated for spot blotch resistance at the seedling stage in a temperature-controlled glasshouse facility at the Queensland Department of Agriculture and Fisheries (QDAF) in the Hermitage Research Facility, Warwick, Queensland, in 2012 and 2013. Barley seeds were sown with three replicates in a randomised complete block design into 10 cm diameter pots, filled with Searles® premium potting mix. The seedlings were inoculated 12 days after sowing, at approximately the Zadok scale growth stage 13 (Z13) (Zadoks et al. 1974), as described by Meldrum et al. (2004). The conidial suspensions were applied at a rate of 3 mL/pot using a spray paint gun, spraying with even passes from all sides. Plants were then transferred into an incubation chamber maintained at 99% humidity using an ultrasonic humidifier. Seedlings were incubated for 24 h at 19ºC (± 4ºC) in the dark, then transferred back to the glasshouse for disease development. Phenotypic infection responses were recorded 12 days later (Z15 growth stage).

**Fig. 1:**
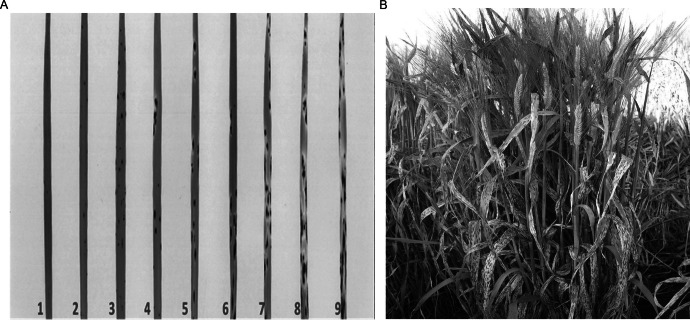
Spot Blotch Disease Reactions. **A.** The 1–9 scale for spot blotch disease reactions in seedlings, where 1 = resistant, and 9 = susceptible. **B.** An elite barley line showing susceptibility to spot blotch in the field

Meldrum et al. 2004) post inoculation using the 1–9 scale, where 1 = resistant and 9 = susceptible (Fig. 1).

### Field screening for adult-plant resistance

BP1 and BP2 were evaluated for spot blotch resistance under field conditions at the Queensland Department of Agriculture and Fisheries (QDAF) in the Redlands Research Facility, Queensland in 2012 and 2013. In both years, the populations were organised in a resolvable incomplete block augmented design with two replicates of the commercial cultivars (Supp. Table 1). The mycelial broth was prepared by blending the V8 agar mycelium until a smooth consistency was reached and then the broth was strained through a fine mesh. Uptima Tween® 20 detergent was also added to aid adhesion to the leaves. The disease spreader blocks were pre-mist irrigated and the mycelial broth applied using a battery powered backpack. Plants were immediately covered with a tarpaulin for approximately 15 h with regular short interval misting irrigation was conducted. These spreader rows became heavily infected after two months and were then used to inoculate the designated spreader rows within the hill plot screening nursery. Infected straw was frequently and evenly spread to inoculate the spreader rows in conjunction with sprinkler irrigation. Genotype responses to the spot blotch infection were recorded once sufficient differentiation was obtained among a known set of reference varieties.

### Genotyping and marker curation

From the BP1 and BP2 populations, 337 unique barley lines were genotyped with DArT-seq markers using the Barley GBS 1.0 platform (http://www.diversityarrays.com), returning a total of 21,364 single nucleotide polymorphic (SNP) markers. SNP marker data was curated by excluding markers with more than 30% missing SNP calls and a minor allele frequency (MAF) of less than or equal to 5% in R (R Development Core Team 2022). Following curation, a total of 4,767 high-quality polymorphic SNP markers were retained and used for all downstream genomic analyses.

### Population structure

Genetic distances were calculated between individuals using the modified Roger’s distances (Wright 1984). The resultant genetic distance matrix was then used in a principal component analysis (PCA) to visualise the population structure. Genotypes were assigned to groups for further exploration of the population structure by applying the k-means clustering approach to the first two principal components in R (Hartigan and Wong 1979, Voss-Fels et al. 2015).

### Phenotypic data analysis

A linear mixed model was fit to the disease response data to account for spatial variation within the screening nurseries using ASReml-R (Butler et al. 2009). To obtain best linear unbiased estimates (BLUEs), genotype was fitted as a fixed effect and replicate as a random effect, while a spatial correlation variance structure was applied to the residual variance, using the auto-regressive one variance model (AR1), where genotypes were indexed by their column and row position. Predictions of genotype performance for disease response were estimated by generating BLUEs. Phenotypic correlations between disease response BLUEs obtained across four environments were calculated using the Pearson correlation coefficient in the “PerformanceAnalytics” (v.1.4.3541) package (Peterson et al. 2018) in R.

### QTL discovery using a single-marker GWAS approach

GWAS was performed in R using the GenABEL (v1.8–0) package (Aulchenko et al. 2007) for seedling and adult plant BLUEs across the experiments. The mixed linear model was adjusted for population stratification by including a kinship matrix to account for any variation created by the relatedness between genotype pairs and the first two principal components to account for population structure. For each of the four datasets, a Manhattan plot was created to display the marker-trait associations across the seven chromosomes and the Bonferroni method (Sidak 1967) (p < 0.05) was used to calculate the threshold value -log_10_(*p*) ≥ 4.78 to identify the statistically significant associations.

### Haplotype-based mapping using the local GEBV method

The local genomic estimated breeding value (Local GEBV) approach was performed using the SelectionTools 19.3 package in R (Voss-Fels et al. 2019). SNP markers (4,767) used in the single-marker GWAS approach were further filtered, removing those that could not be mapped to a chromosome and/or positioned within the chromosome, resulting in 3,010 markers. SNP markers were assigned to LD blocks by grouping markers based on pairwise r^2^ with a minimum LD threshold of r^2^ = 0.5. Prior to assigning LD blocks, the pairwise r^2^ values between SNP markers were first calculated across each chromosome, allowing selection of the highest LD among adjacent pairs of markers within each chromosome. For each data set, genome-wide single-marker effects were first calculated simultaneously using ridge-regression best linear unbiased predictions (RR-BLUP; Meuwissen et al. 2001). The initial RR-BLUP model ignores SNP effects of neighbouring markers in LD with the marker being estimated, to ensure that the SNP effects are not overestimated. This model attribute is important in the LD block effect calculation, ensuring single-marker effects are only captured once. Individual SNP effects within each LD block was then summed to calculate the individual haplotype effects within each LD block across the genome. The variance between haplotype effects within each LD block was then estimated and plotted as a Manhattan plot, where blocks with high variance were considered most associated with the trait (Voss-Fels et al. 2019).

The top 20 LD blocks for each environment were selected based on the highest variance. The allele contributing resistance or susceptibility at each marker within these LD blocks was determined along with the overall block effects, thus allowing blocks to be assigned to groups as either resistance or susceptibility blocks. Due to having a high number of lines within each nursery, the 2013 datasets were selected for downstream haplotype analysis focusing on the LD blocks with the highest variance for haplotype effects identified on chromosome 3H and 7H.

### Haplotype network analysis

Haplotype network analysis was performed for the block identified on chromosome 3H. TCS genealogies between haplotype variants (Clement et al. 2000), were calculated using PopART (http://popart.otago.ac.nz.; Leigh and Bryant 2015). The network nodes were coloured according to the average disease rating in the respective haplotype groups.

## Results

### Spot blotch disease varies across nurseries

**Fig. 2:**
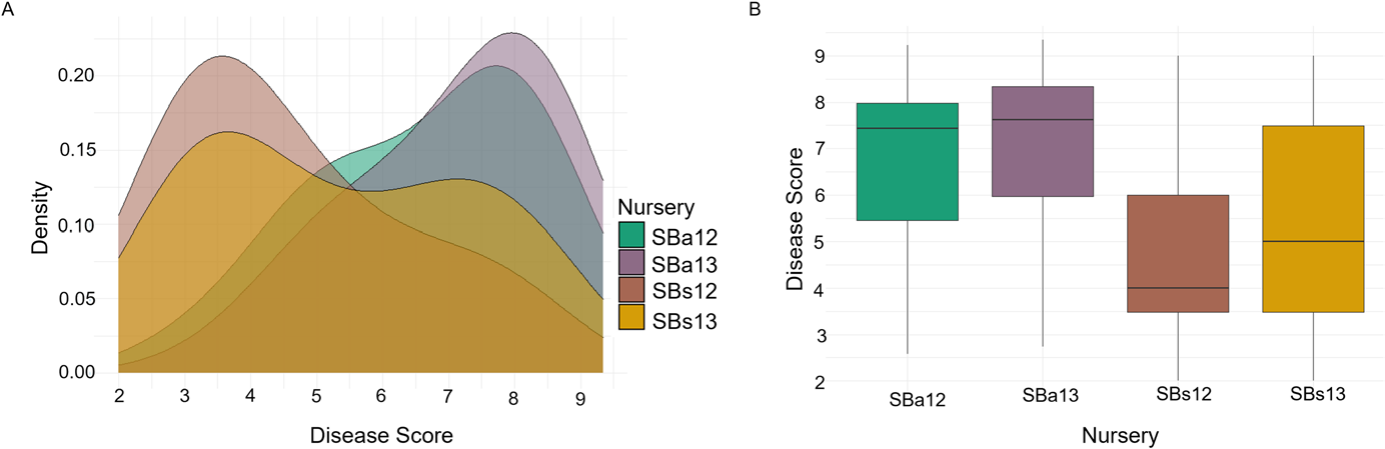
Spot Blotch Disease Response across Nurseries **A.** Frequency distribution and **B.** boxplots of spot blotch disease response, where 1 is resistant and 9 is susceptible, across adult (SBa12 and SBa13) and seedling nurseries (SBs12 and SBs13)

A high degree of variation in disease responses was observed for BP1 and BP2 at all growth stages (Fig. 2A). Field nurseries assessing adult-plant stage resistance (SBa12 and SBa13) had a higher frequency of susceptible disease response scores than the seedling glasshouse nurseries (SBs12 and SBs13; Fig. 2B). This trend was consistently observed in the control lines, with the exception of the resistance reference controls (Supp. Table 1.)

**Fig. 3:**
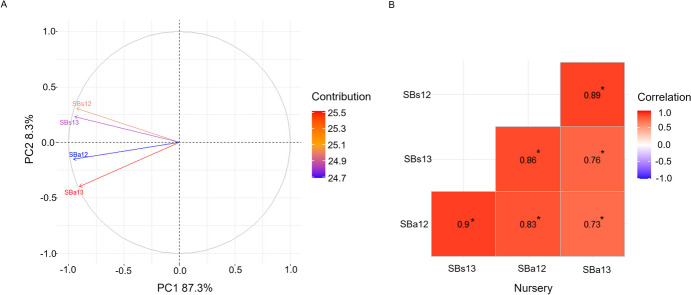
Phenotypic Relationships between Spot Blotch Nurseries. **A.** Principal component analysis, displaying PC1 plotted against PC2, of spot blotch BLUEs across the four nurseries. **B.** Pearson correlation coefficient (*r*) between each of the nurseries, where * represents p < 0.05

All nurseries appear related and trend in the same direction for principal component (PC) 1, which accounts for 87.3% of the variation (Fig. 3A). PC2, accounting for almost the remainder of the variation, demonstrates a clear separation between the adult and seedling nurseries. A statistically significant (p < 0.05) and strong positive correlation was observed between both seedling nurseries (SBs12 and SBs13; *r* = 0.89; Fig. 3B). Similarly, a high correlation was observed between the disease responses from the adult plant nurseries (SBa12 and SBa13; *r* = 0.89). Interestingly, the correlation between seedling and adult phenotypes was lower, both in 2012 (SBs12 and SBa12; *r* = 0.67) and in 2013 (SBs13 and SBa13; *r* = 0.69), but still statistically significant.

### Population structure and genetic relatedness

**Fig. 4:**
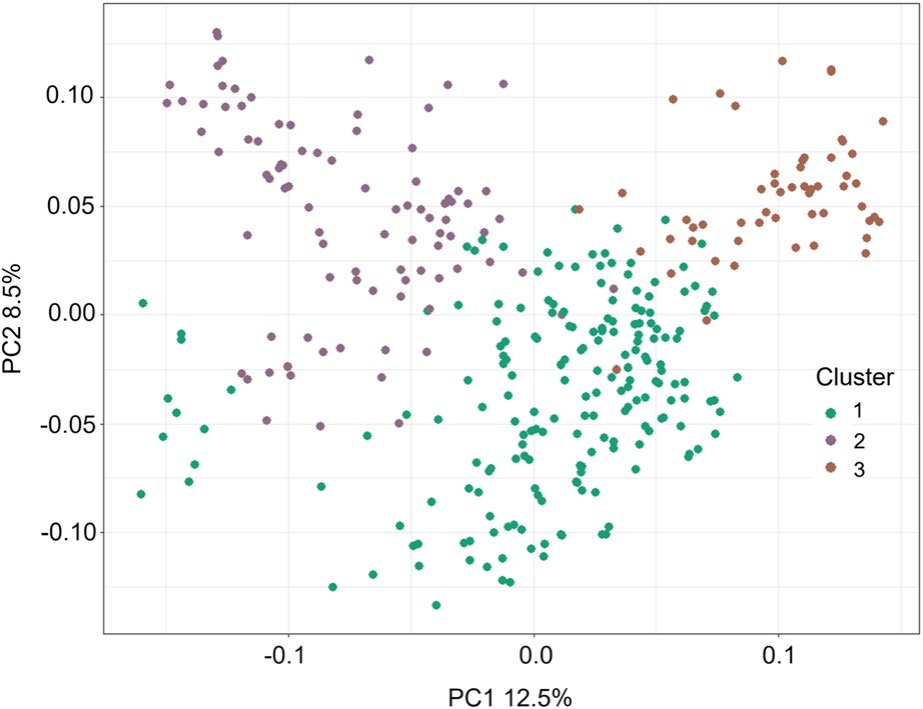
Principal Component Analysis (PCA) of genetic distances among the barley breeding panel, illustrating population structure. The three clusters, represented by distinct colours (clusters 1, 2, and 3), were identified using k-means clustering and are plotted based on the first two principal components (PC1 and PC2)

The PCA of the genetic distance between individuals in the breeding populations revealed three main clusters (Fig. 4). PC1 accounts for 12.9% of the variation and PC2 accounting for 8.9%, suggesting minimal population structure within the breeding population. Genotypes within the three clusters were investigated, where cluster 1 contained 106 lines (BP1 = 32, BP2 = 74; 8 common), cluster 2 contained 114 lines (BP1 = 57, BP2 = 57; 6 common), and cluster 3 contained 90 lines (BP1 = 45, BP2 = 45; 13 common), yet no definitive patterns could be identified in the absence of pedigree information (Fig. 4).

### Single-marker genome-wide association mapping

**Fig. 5:**
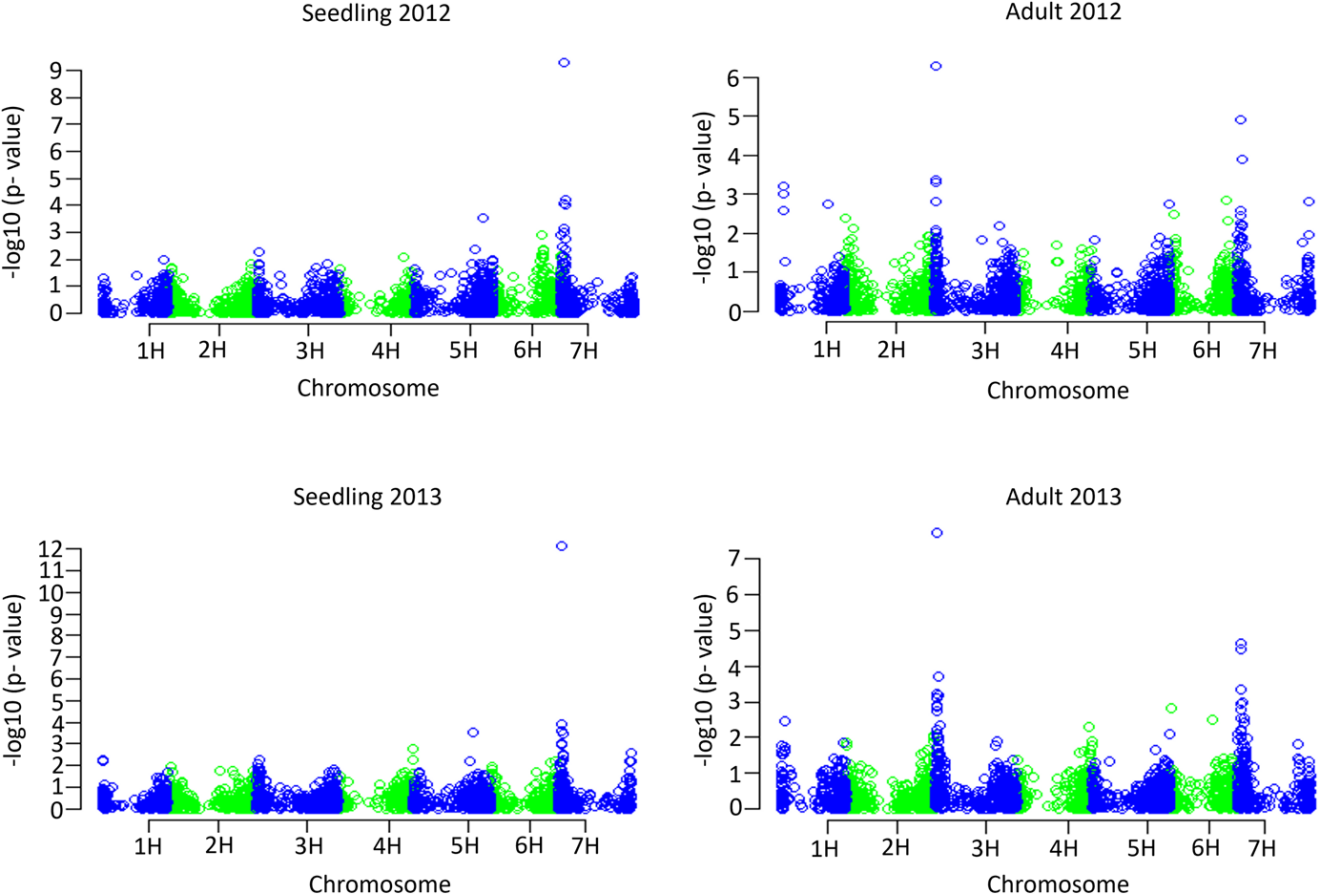
Manhattan plots displaying significant marker-trait associations for spot blotch resistance in seedling and adult plant assays conducted in 2012 and 2013. A significance threshold of -log_10_(*p*) ≥ 4.78 was adopted based on the Bonferroni method (*P* < 0.05)

A total of 24 markers were significantly associated with SB disease susceptibility across the four nurseries and detected on chromosomes 1H, 3H, 5H and 7H (Fig. 5; Supplementary Table 2), half of which were detected at the seedling stage and the remaining half at the adult plant stage. Of the QTLs identified, some were environment and growth stage specific, for example two QTLs on 1H were identified in 2012 for adult plant stage resistance. In contrast, the three QTLs on 7H at markers 3,398,217, 3,256,980 and 3,259,148 were detected across all four environments and growth stages. Similarly, the QTL detected on 3H at marker 3,665,142 was strongly associated to adult plant stage resistance across both years (Fig. 5).

### LD blocks associated with spot blotch response

**Fig. 6:**
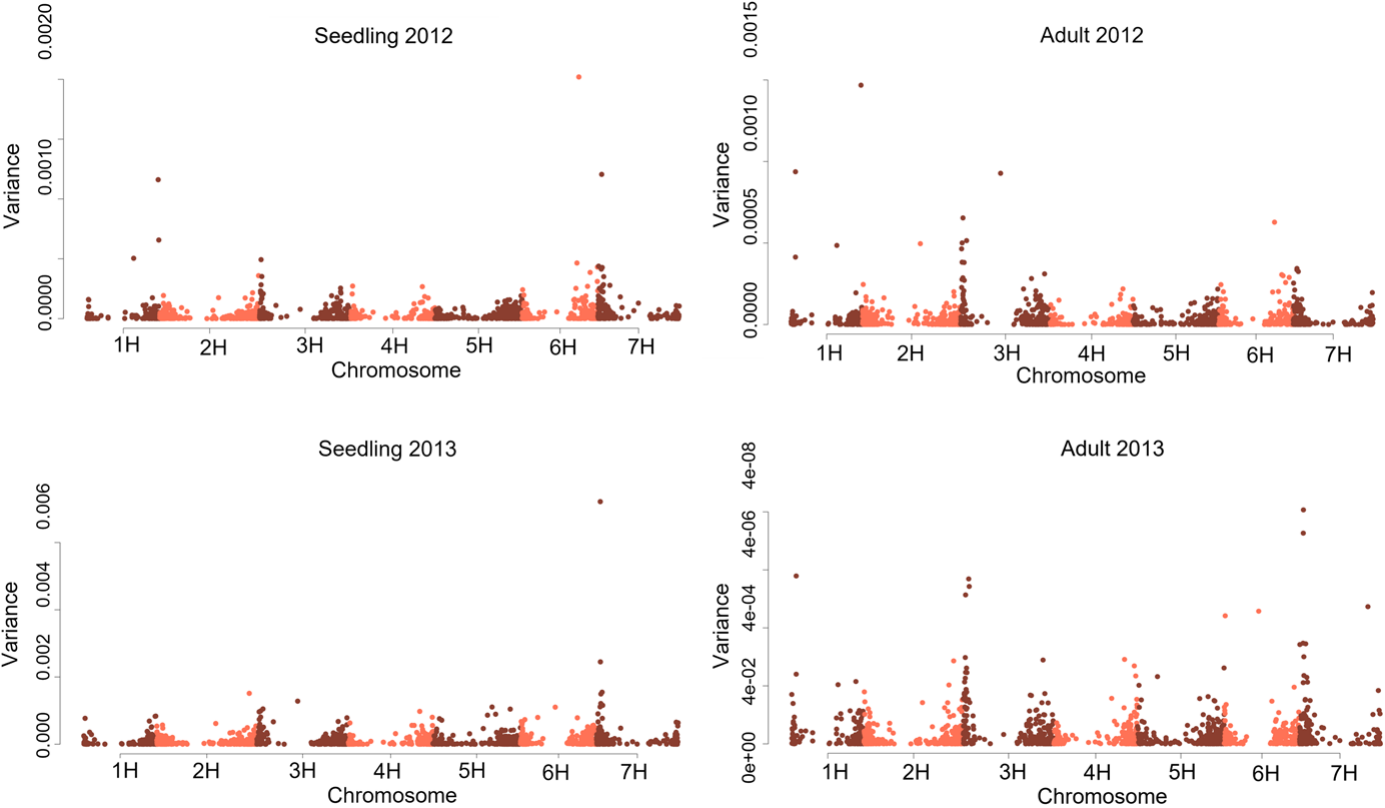
Manhattan plots displaying LD blocks of markers with high variances for seedling and adult plant resistance to spot blotch. Blocks were identified using the local GEBV approach to assess haplotype-trait associations in four nurseries across two years

Characterisation of the LD structure across chromosomes identified 1,610 LD blocks. For each seedling and adult plant nursery dataset, the top 20 LD blocks explaining the highest proportion of variance for haplotype effects were selected (Fig. 6), resulting in 80 blocks in total. Among these, 55 blocks were shared across environments. Notably, block b001471 on chromosome 7H was consistently detected across all environments and growth stages (Supplementary Table 3), with a high proportion of variance for haplotype effects. This block also included marker 3256980, which was similarly identified across all environments in the single-marker analysis. Another block, b000531, was detected across methodologies and nurseries but comprised only a single marker.

Interestingly, block b000552 on chromosome 3H consistently explained a high proportion of variance across three environments (SBa12, SBs13 and SBa13; Supplementary Table 3) but was not identified in the single-marker analysis. Additional QTL were identified using the local GEBV method but were absent in the single-marker approach, such as blocks on 1H in SBs12 and SBa13, and blocks on the short and long arms of chromosome 6H. A higher proportion of variance for haplotype effects was observed in the seedling nurseries conducted under controlled conditions compared to the adult plant nurseries grown in field environments.

### Haplotype analyses and allele stacking

**Fig. 7:**
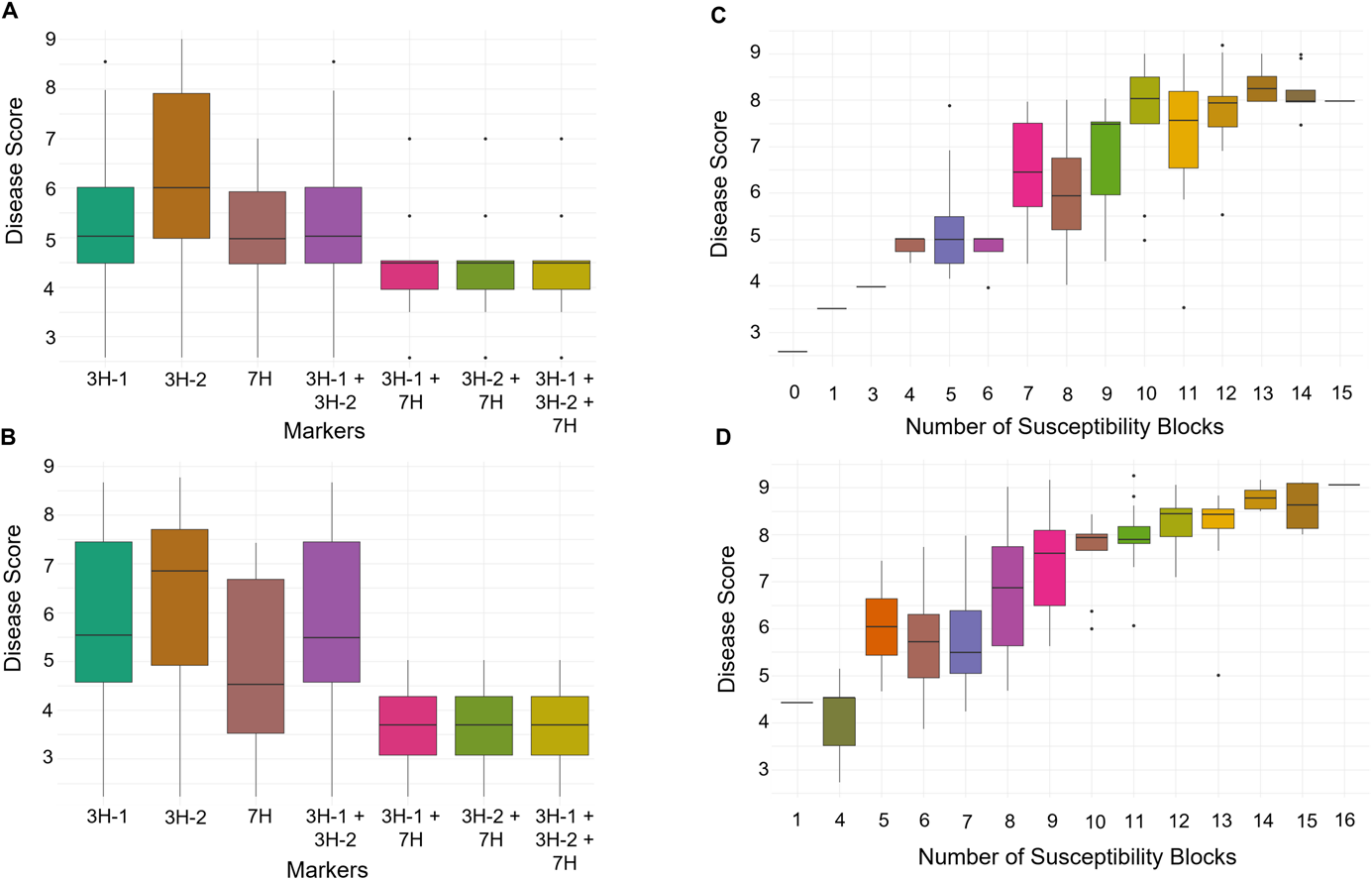
The spot blotch disease score for lines with and without major susceptibility alleles identified in the adult plant nurseries across 2012 and 2013. **A.** Disease score in SBa12 nursery for lines without the haplotypes conferring susceptibility at major blocks identified on 3H (3H-1 and 3H-2) and 7H. **B.** Disease score in SBa13 nursery for lines without the haplotypes conferring susceptibility at major blocks identified on 3H (3H-1 and 3H-2) and 7H. **C.** Regression of phenotypic disease score from SBa12 nursery on the number of susceptibility haplotypes held by breeding population individuals. **D.** Regression of phenotypic disease score from SBa13 nursery on the number of susceptibility haplotypes held by breeding population individuals with the respective disease score

Of the 80 LD blocks identified, three were selected for further haplotype analysis due to their high variance for haplotype effects and their consistent detection across multiple environments. This included b000531 (3H-1) and b000552 (3H-2) both located on chromosome 3H, as well as b001471 on 7H. Both b000531 and b001471 contained only 1 marker and were also identified in the single-marker analysis (Supplementary Table 2). With all three blocks having an effect in the adult plant nurseries, the absence of susceptibility haplotypes at each block and effect on disease score was explored across both nurseries (Fig. 7). Consistent variance for disease scores was observed across SBa12 and SBa13, where large variance in disease score was observed when susceptibility alleles were absent in only one of the three blocks or in both 3H blocks (Fig. 7A and B). Interestingly, the largest reduction in disease score and variance was achieved when the 7H susceptibility haplotype was absent in combination with either or both 3H block’s susceptibility haplotypes. This suggests that minimising susceptibility haplotypes at the 7H block in combination with reducing other major genetic contributors to susceptibility is highly beneficial. In the breeding panel, 135 of the 337 genotypes carried the 7H susceptibility allele**,** demonstrating the population is enriched with the resistance allele.

The compounding effect of susceptibility haploblocks on disease score was explored in the SBa12 and SBa13 adult plant nurseries, and consistent results were observed across nurseries (Fig. 7C, D), where disease scores increased in a linear trend with the addition of susceptibility blocks. Very high levels of disease susceptibility were reached and plateaued once 13 and 14 susceptibility blocks were present within an individual in SBa12 and SBa13, respectively. Interestingly, there were a reasonable number of individuals within each nursery with greater than 13 susceptibility haploblocks. Yet, this is consistent with the general level of spot blotch susceptibility within Australian barley cultivars and is further supported by the limited number of individuals with four or less of the major susceptibility haplotypes across both nurseries. Individuals with four or less susceptibility haplotypes displayed moderately resistant to resistant disease scores across both adult plant nurseries (Fig. 7C, D).

**Fig. 8:**
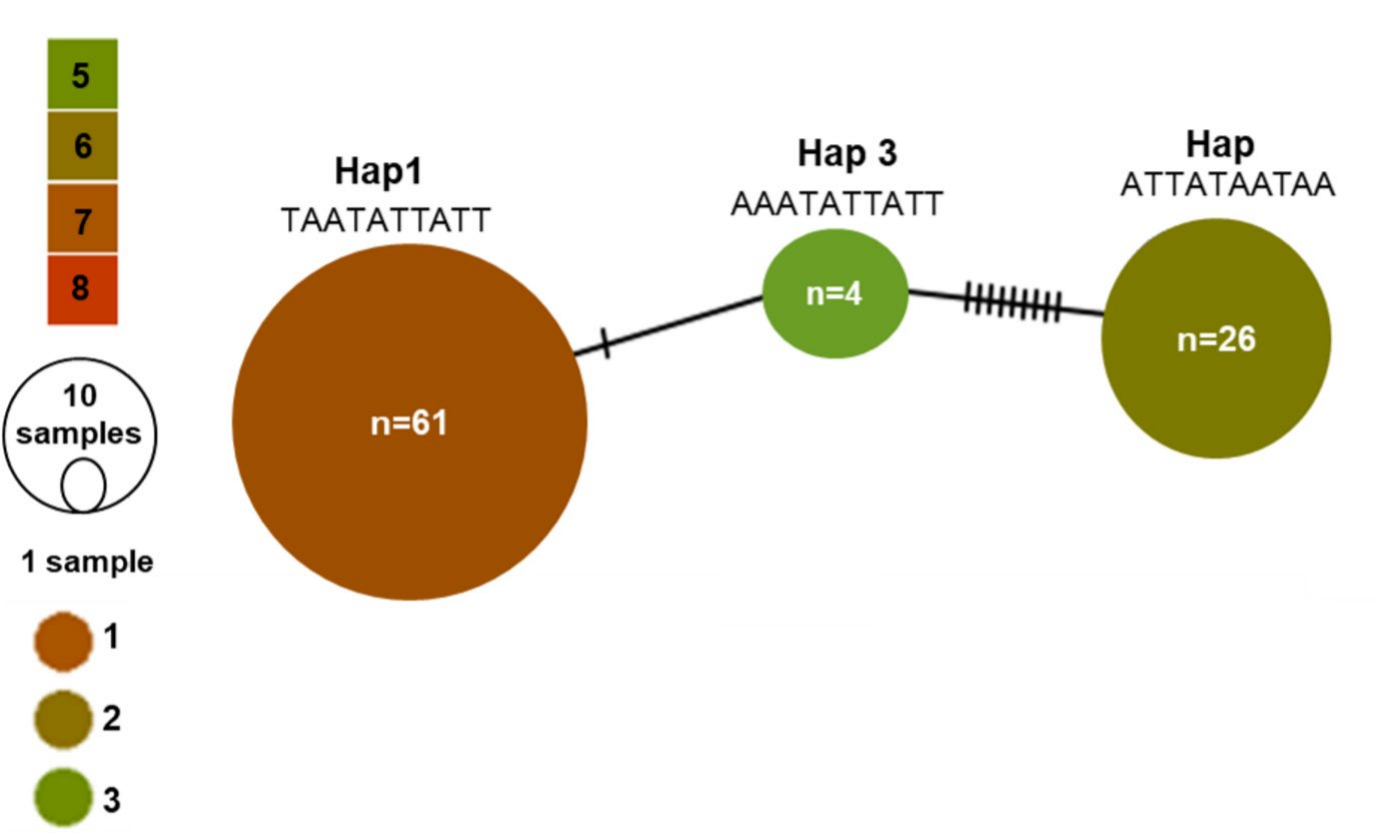
Haplotype network illustrating three haplotype variants for the 3H block in the NRBB program. Node size is proportional to the number of lines (n) carrying each haplotype variant, while node color represents the adult plant disease response observed in the SBa12 environment

Of the three major blocks identified, only b000552 on chromosome 3H, contained more than one marker and was therefore selected to further explore haplotype variation through network analysis. The high proportion of variance explained by the block along with its consistent effects across years at the adult growth stage also makes it an ideal candidate. Three haplotype variants for the block were evident (Fig. 8), where haplotype 1 (Hap 1) contained 61 genotypes, consisting of a mixture of commercial varieties and NRBBP lines, with a mean disease response of 7.41. In contrast, haplotype 2 (Hap 2) contained 26 lines, predominately originating from the NRBBP and North Dakota and had a mean of 6.52. The smallest haplotype group (Hap 3) consisted of only four lines that all originated from the NRBBP and had a mean disease response of 5.38, the lowest of all haplotypes (Fig. 8; Supplementary Table 4).

## Discussion

### Useful sources of spot blotch resistance

Cultivars with durable genetic resistance to spot blotch are the most effective method for controlling the disease. While Australian cultivars are known to be susceptible to spot blotch, our understanding of whether Australian germplasm contains the previously identified genetic sources of resistance remains limited. This study is the first to examine the genetic architecture of spot botch susceptibility in Australian breeding germplasm, using both single-marker and haplotype-based mapping approaches. We evaluated two breeding populations from the NRBBP against the same spot blotch pathotype (SB61) across four environments. Two of these environments focused on disease response in seedling glasshouse assays, while the other two assessed adult plants grown under field conditions. We found strong phenotypic correlations between environments, particularly between those assessing similar growth stages and growth conditions. Our results demonstrate that most of the germplasm evaluated was moderately to highly susceptible to spot blotch, especially when assessed as adult plants under field conditions. However, variation in disease response was observed in both seedling glasshouse and adult field assays, indicating the presence of genetic resistance in Australian germplasm. We discuss the nature of these sources of resistance and how new technologies can be applied to improve selection in breeding.

### Genomic regions associated with spot blotch response

Two breeding populations from the NRBBP were evaluated as part of this study, yet the variation driving the three clusters in the PCA (Fig. 5) could not be explained by the two populations. This is likely a result of the two populations having shared ancestry and thus moderate relatedness due to being part of the same breeding program. However, limited pedigree information is available to validate the assumption of relatedness between the populations. Across both mapping approaches, we identified 29 chromosomal regions associated with spot blotch disease susceptibility, with reasonable consistency of results across methodologies. The highly significant QTL identified on chromosome 7H using the single-marker approach was also detected using the haplotype-based approach (b001471). Furthermore, the 3H QTL identified in three out of the four environments was also identified across methods (b000531, 3H-1). Notably, the local GEBV approach identified additional haploblocks on 1H, 3H and 6H, which were not detected using the single-marker method, but were detected across environments. Our results suggest that the local GEBV approach may offer an advantage in this context, as it identifies the same significant QTLs as the single-marker approach, while also detecting novel LD blocks that were not captured by the latter. Yet, it is important to note that this approach requires further validation to ensure that the novel QTLs detected are not false positives. However, the moderate level of LD within the population, likely a result of inbreeding and selection, plausibly provides greater power to detect significant marker trait associations across the haplotypes (Nielsen et al. 2004). Further, tightly linked loci generally undergo less recombination events, therefore this haplotype-based approach can be considered a more realistic representation of the effects when using significant marker-trait associations in breeding selection and progeny development.

The haploblocks detected on chromosomes 3H and 7H were in close proximity with QTL reported in the literature and catalogued resistance gene *Rcs5*, respectively (Steffenson et al. 1996; Bilgic et al. 2005; Bovill et al. 2010; Roy et al. 2010; Zhou and Steffenson 2013). More specifically, these two blocks appear to co-locate with two seedling resistance QTL (SRT-SB54-3 and SRT-ICSB3-10) reported by Visioni et al. (2020) for two differing pathotypes that potentially co-locate with *Rbs1*. Interestingly, our study observed resistance at both loci for seedling and adult plant resistance, yet this could be driven by the differing pathotype. *Rcs5* is a major gene located on chromosome 7H and it confers seedling resistance to spot blotch with strong origins in barley germplasm bred by the United States North Dakota barley breeding program (Steffenson et al. 1996). It is well-known that the NRBBP utilised germplasm from the North Dakota breeding program as foundational parents, so it is not unexpected that *Rcs5* is present within this population, but this is the first study to provide confirmation. The additional block on chromosome 1H identified using the Local GEBV method also coincide with *Rcs6* and several other reported QTL in the region (Bilgic et al. 2006; Grewal et al. 2012; Zhou and Steffenson 2013; Haas et al. 2016). Similarly, the block identified on the short arm of chromosome 6H may also align with *Rbs7*, however the block on the long arm of 6H is likely novel. The differing molecular marker genotyping platforms used in the literature made it challenging to accurately align the QTLs detected in this study, however, based on their approximate position on the chromosome the alignments seem plausible, yet this requires validation.

### Allele stacking to achieve high levels of resistance

The results of this study along with the earlier literature clearly demonstrate that the genetic architecture of spot blotch is highly complex and quantitative in nature. Although some previous success has been achieved using marker-assisted selection (MAS; Kottapalli et al. 2010; Castro et al. 2012), to achieve a durable level of resistance across growth stages, a multi-allele stacking or whole genome selection approach could be the most effective breeding strategy. Outcomes of this study support a quantitative approach, where high disease scores and variance were observed when each of the three major susceptibility blocks were absent on an individual basis, or when both of the 3H susceptibility blocks were absent. Interestingly, when the 7H block was combined with either or both the 3H blocks, a substantial reduction in disease score and variance were observed (Fig. 7A, B). Based on its chromosomal location, we hypothesise that the 7H block identified in our study corresponds to *Rcs5*, a major resistance gene widely recognised across the global barley populations and commonly found in germplasm from North Dakota. The *Rcs5* region is well established as a key contributor to all-stage seedling resistance; however, its effectiveness in conferring adult-plant resistance has shown variability. Previous studies suggest this variability is influenced by significant gene-by-environment and/or gene-by-gene interactions (Bovill et al. 2010). In our study, the consistent results from the two adult plant nurseries indicate no evidence of gene-by-environment interactions, but a clear gene-by-gene interaction was observed when the 7H block was combined with other major sources of adult-plant resistance. This highlights that leveraging a combinatorial strategy may be the most effective way to utilise the *Rsc5* region for adult-plant resistance. Furthermore, our global haploblock stacking analysis (Fig. 7C, 7D) underscores the importance of reducing susceptibility haplotypes through a quantitative breeding approach to achieve rapid and durable spot blotch resistance in Australian barley.

A significant proportion of the breeding population in the NRBBP contains susceptibility haplotypes at both the 3H and 7H blocks, despite active selection for spot blotch disease resistance within the breeding program. Since breeding programs routinely select for numerous traits such as yield, multiple disease resistance and end-use grain quality traits, and either explicitly or innately apply a selection weighting, it is possible that the major haploblocks under selection could have pleiotropic effects influencing multiple traits. Previous research suggests that key spot blotch susceptibility genes may have negative pleiotropic effects. Specifically, Scs6 and Rcs5 are reported to be negatively associated with resistance to powdery mildew and leaf rust, respectively, and both of these genes are likely present in the NRBBP. As a result, careful consideration needs to be given before strong selection against the major susceptibility haploblocks identified in this study. Yet, our results demonstrate that genetic resistance is highly quantitative, and as such a reasonable level of disease resistance could still be achieved in the presence of the major susceptibility blocks through the minimisation of other susceptibility alleles (Fig. 7C, D). Further research that simulates the presence of the major susceptibility blocks identified on chromosomes 1H, 3H, 6H, and 7H, and explores the gradual reduction of alternative susceptibility haplotypes, could provide valuable insights into the number of haplotypes needed to achieve sufficient levels of resistance for breeding purposes. This potential negative linkage highlights the importance of continually exploring diverse germplasm for novel sources of resistance to spot blotch and using new breeding technologies for rapid introgression. Our study illustrates the potential of the local GEBV approach in providing a detailed representation of the genetic architecture of quantitative traits and highlights its usefulness for breeders in selecting for or against multiple haplotypes.

This study represents one of the first to investigations into spot blotch resistance within an Australian barely breeding program, revealing that significant variation for resistance exists within Australian germplasm, which breeders can leverage for cultivar improvement. This finding is particularly impactful for the industry, as alternative methods like introgressing diverse resistant germplasm are time-consuming and can take years to integrate into breeding programs. Our research further underscores the quantitative genetic nature of spot blotch resistance and, for the first time, explores susceptibility haplotypes within LD blocks, moving beyond the traditional single-marker mapping approach. We demonstrate the power of this haplotype-based method by identifying consistent marker-trait associations and novel haploblocks that were not detected by the single-marker approach or in existing literature. Additionally, we highlight the potential of a haplotype stacking to select against major susceptibility haplotypes, showing that resistance can be achieved by minimising the number of susceptibility blocks, regardless of the presence or absence of major haploblocks. However, given the negative pleotropic effects previously reported for spot blotch resistance, careful consideration is needed when selecting against major susceptibility haplotypes, and a holistic trait selection strategy is likely the most effective approach. In a time when developing spot blotch resistant cultivars is more critical than ever, our study provides valuable insight for Australian barley breeding and introduces an innovative, yet practical, approach that can be directly applied within the industry.

## Supplementary Information

Below is the link to the electronic supplementary material.

Supplementary file1 (XLSX 25 KB)

## Data Availability

The datasets generated during and/or analysed during the current study are available from the corresponding author on reasonable request.
